# Possible utility of neutrophil/lymphocyte ratio as a predictor of suicidal risk in mood disorders

**DOI:** 10.1192/j.eurpsy.2022.2169

**Published:** 2022-09-01

**Authors:** W. Flamini, S. Torrigiani, F. Mucci, T. Ivaldi, D. Marazziti, L. Dell’Osso

**Affiliations:** 1 University of Pisa, Department Of Clinical And Experimental Medicine, Section Of Psychiatry, Pisa, Italy; 2 University of Siena, Department Of Biotechnology, Chemistry And Pharmacy, Siena, Italy

**Keywords:** Mood disorders, neutrophil/lymphocyte ratio, suicide risk, NLR

## Abstract

**Introduction:**

Correlations between neutrophil/lymphocyte, platelet/lymphocyte, and monocyte/lymphocyte ratios (NLR, PLR, and MLR, respectively) and psychopathological and clinical variables in the context of mood disorders are increasingly emerging in international scientific literature, being the former one of the most studied. The estimation of suicidal risk associated to affective disorders could benefit from such rapidly and easily available biomarker of inflammation, if significant in this regard.

**Objectives:**

The present review would like to focus on any existing correlations between NLR and suicidal risk in patients with mood disorders.

**Methods:**

We sourced articles on the topic found in major scientific literature databases, combining the keywords “neutrophil/lymphocyte ratio”, “NLR”, “mood disorders”, “major depressive disorder”, “bipolar disorder” and “suicide risk”.

**Results:**

There are congruent findings of significantly higher NLR values in depressed patients attempting suicide than in depressed patients with no suicidal behaviors or healthy controls. In addition, violent means appear typical in this subgroup of depressed suicidal patients, suggesting that patients with higher levels of NLR are at risk of attempting suicide and to be successful in self-harming. Similar results have been found in patients with bipolar disorder, showing a positive correlation between NRL and suicide risk, evaluated by the Suicide Behaviors Questionnaire-Revised (SBQ-R). Moreover, in patients with a positive family history for suicide attempts, NRL was found to be a significant positive predictor of suicide risk.

**Conclusions:**

These findings, although limited, support the notion that NLR might be a useful marker for suicide vulnerability in both BD and MDD patients.

**Disclosure:**

No significant relationships.

